# The assessment of geriatric malnutrition, geriatric depression and associated co-morbidities among forcibly displaced Myanmar nationals in Bangladesh

**DOI:** 10.1017/S1368980023001556

**Published:** 2023-10

**Authors:** Mohammad Morshad Alam, Rajib Das, Afrin Ahmed Clara, Faroque Md Mohsin, Md Anwar Hossain Rumi, Abrar Wahab, Md Abeed Hasan, Mohammad Delwer Hossain Hawlader

**Affiliations:** 1 Department of Public Health, North South University, Dhaka 1213, Bangladesh; 2 Health Nutrition and Population Global Practice, The World Bank, Dhaka, Bangladesh; 3 International Organization for Migration, Dhaka, Bangladesh; 4 Directorate General of Health Services, Ministry of Health and Family Welfare, Dhaka, Bangladesh; 5 Save the Children Bangladesh, Dhaka, Bangladesh

**Keywords:** FDMN, Malnutrition, Depression, Co-morbidities, Prevalence

## Abstract

**Objective:**

To assess the nutritional status and depression of the elderly forcibly displaced Myanmar nationals (FDMN) in Bangladesh and determine the associated factors of geriatric depression (GD).

**Design:**

This was a community-based, cross-sectional study among elderly FDMN. The Mini Nutritional Assessment Short-Form (MNA@-SF) and Geriatric Depression Scale Short-Form (GDS-15 SF) were used to determine malnutrition and GD, respectively.

**Setting:**

The study was conducted between November 2021 and March 2022 in Kutupalong Refugee Camp, Cox’s Bazar, Bangladesh.

**Participants:**

The study participants were elderly FDMN aged ≥ 60 years (*n* 430).

**Results:**

The mean age and BMI were 71·7(±7·8) years and 21·94(±2·6) kg/m^2^, respectively. There was a high prevalence of self-reported diabetes mellitus (32·1 %), hypertension (26·7 %), hypotension (20 %), skin diseases (28·4 %) and chronic obstructive pulmonary disease (16·5 %). The prevalence of malnutrition was 25·3 %, and another 29·1 % were at risk. The prevalence of GD was 57·9 %, and co-occurrences of GD and malnutrition were seen in 17·5 % of participants. GD was significantly higher among elderly people with malnutrition (adjusted OR, AOR = 1·71, 95 % CI: 1·01, 2·89). FDMN aged ≥ 80 years were at higher risk of GD (AOR = 1·84, 95 % CI: 1·01, 3·37), and having fewer than five members in the household was an independent predictor of GD. Diabetes mellitus (AOR = 1·95, 95 % CI: 1·24, 3·08) and hypotension (AOR = 2·17, 95 % CI: 1·25, 2·78) were also significantly associated with an increased risk of GD.

**Conclusion:**

A high prevalence of GD and malnutrition was observed among elderly FDMN in Bangladesh. The agencies working in Cox’s Bazar should focus on geriatric malnutrition and GD for the improvement of the health situation of FDMN in Bangladesh.

Older people are more vulnerable to various non-communicable diseases (NCD), that is, mental health issues, malnutrition, decreased functional activity, and loss of skills, and they are also vulnerable to infectious diseases^(1)^. Among older adults, depression-related symptoms are more or less similar to those of other chronic diseases, resulting in challenging diagnosis and delays in treatment^(2)^. Worldwide, about 10 % to 20 % of older people are estimated to live with depressive symptoms, which may increase the risk of health complications, suicide and overall mortality^(3,4)^. Many researchers have discussed the correlation between malnutrition and depression at an older age, and several studies showed an interdependent statistical relationship between these geriatric health problems^(5)^. According to the WHO report of 2017, approximately 15 % of older-aged (≥ 60 years) people suffer from various mental disorders, which accounts for 6·6 % of the total disability^(6)^.

Malnutrition and undernutrition have already merited attention like other typical and highly prevalent geriatric syndromes, that is, dementia, incontinence, delirium, falls, loss of function, etc.^(7)^. Malnutrition is the result of deficiencies or imbalances in nutrient intake and addresses three broad conditions, including undernutrition, stunting and underweight. Undernutrition indicates low weight-for-height and is defined as not consuming enough nutrients and energy to meet one’s needs for maintaining good health^(8)^. Among the older people, a decrease in food intake combined with increased nutrition needs due to various illnesses made them vulnerable to malnutrition^(9)^. Previous research has established malnutrition as a risk factor for increased morbidity and mortality in diseased conditions and as having a negative impact on recovery^(10)^. Some other studies also discussed the interrelationship between geriatric malnutrition and depression. The studies also revealed an interdependent association between these two geriatric health problems^(5)^.

Older adults affected by war, displacement or natural disasters usually face more physiological and psychological issues. Therefore, a higher prevalence of depressive symptoms and other chronic diseases are seen among older adults compared to the general population^(11,12)^. The forcibly displaced Myanmar nationals (FDMN) are Muslim minority people of Myanmar who had to leave their motherland, Rakhine State, due to violence and genocides by the Myanmar Army, and they relocated in Rohingya camps of Cox’s Bazar, Bangladesh^(13,14)^. These camps are very densely populated, with about 40 000 people per square kilometre, and there is an unavailability of clean water, food, sanitation and basic health services^(15,16)^. Recent analysis has suggested a high prevalence of various NCD such as hypertension, diabetes and chronic obstructive pulmonary disease (COPD)^(6,17)^

According to the estimation of the United Nations High Commissioner for Refugees (UNHCR), more than 31 000 elderly FDMN (≥ 60 years of age) live in the Rohingya camps of Cox’s Bazar^(18)^. Due to various health-related challenges in Rohingya camps, the burden of NCD, infectious diseases and poor hygiene practices have created a critical situation for older FDMN^(19)^. Moreover, before coming to Bangladesh, these people experienced the brutality of the military, which severely impacted their eating patterns, difficulty concentrating and other mental health problems^(20)^. There are a number of scales available for screening geriatric depression (GD) and malnutrition in both community and hospital settings. Among those scales, the Geriatric Depression Scale Short-Form (GDS-15 SF) and the Mini Nutritional Assessment Short-Form (MNA@-SF) are two common and reliable scales^(21–23)^. Although there have been many studies worldwide on malnutrition and the mental health of the elderly population, to our best knowledge, we have found no empirical study regarding the status of geriatric malnutrition and its association with mental health problems, including other co-morbidities, in the context of the FDMN population living in Bangladesh.

Therefore, the aim of this study was to assess geriatric malnutrition and GD among FDMN in the Kutupalong Refugee Camp of Cox’s Bazar, Bangladesh. Moreover, we have also determined the association of GD with geriatric malnutrition, selected co-morbidities and demographic factors.

## Methods

### Study design

This was a community-based, analytical cross-sectional study in Bangladesh. To address objectives within the limitations of time and resources, a cross-sectional study design was selected by the research team.

### Study setting and participants

The study was conducted in the Kutupalong refugee camp of Cox’s Bazar district among FDMN aged ≥ 60 years residing in Bangladesh. The data were collected from November 2021 to March 2022. We have excluded people who were not interested or unable to give an interview, that is, unable to communicate, with a hearing disability, or who were seriously ill.

### Sample size calculation

We have calculated the sample size using the following formula; as the prevalence of malnutrition among FDMN is unknown, we have assumed a 50 % prevalence, CI = 95 %, margin of error = 5 % (0·05) and non-response rate = 25 %:

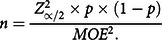




Therefore, after addition of the non-response rate the final estimated sample size was 480.

### Sampling and data collection

There was no list of elderly FDMN with us; therefore, a modified systematic sampling technique was applied to meet the constraints of resources and manpower. In the first step, we selected twenty starting points (households) randomly from various places to select the respondents. The research assistants started household-based data collection from one point in the camp after each of the five household intervals. If the household approached missed the participants maintaining inclusion and exclusion criteria, the research assistants went for the next one and continued until the completion of the desired number of interviews. We have targeted collecting at least twenty datasets from each of those points. If there was an absence of registered documents regarding the essential information of participants, the information was confirmed with the help of enumerators. When there was more than one eligible participant in a household, the oldest case was interviewed. All of the information was collected through a face-to-face interview. Research assistants were local residents of Cox’s Bazar district and also fluent in the Rohingya language.

### Tools and measures

The MNA@-SF is a validated instrument to measure the nutritional status of the elderly population. The MNA® has been used in more than two thousand studies worldwide and is also included in almost all geriatric nutrition textbooks. In this study, we assessed the nutritional status of the elderly FDMN using MNA@-SF, which is a practical tool for the identification of nutritional status^(22)^.

We have measured the depression status of elderly FDMN by using the GDS-15 SF, which historically showed good performance in detecting depression in older adults and has been validated among a wide range of populations^(21)^. A translator was always present during the interview process.

Other than depression and nutritional status, we also targeted determining the status of other co-morbidities such as diabetes, hypertension, COPD and skin diseases in elderly FDMN. The co-morbidity-related information was confirmed through previous verified test reports, medical documents or discussion with family members. The study also analysed various socio-demographic factors that might be associated with GD. Participants who were able to write and read at least one sentence in their mother tongue were categorised as ‘educated’. The BMI was calculated using the respondents’ weight in kg divided by the square of their height in metres.

Before the final data collection, the English version of our questionnaire was translated into the local Bengali language and then back-translated again to English by research assistants to ensure consistency. The local version of the questionnaire was piloted among twenty elderly Rohingya in Kutupalong Refugee Camp for the finalisation of the tool. The Cronbach’s alpha score was 0·71 for GDS-15 SF and 0·74 for MNA@-SF, which indicates good internal consistency of the local version of the tool.

### Data management and analysis

During the study period, we approached 475 elderly FDMN and were able to conduct 430 complete interviews (response rate: 90·5 %), which were subjected to analysis (incomplete interviews were excluded). The data were entered and cleaned in Microsoft Excel (2013) and analysed using Statistical Package for Social Science (SPSS) software version 25. Descriptive statistics were conducted to assess the distribution of variables using frequencies, proportions, pie diagrams, bar diagrams, etc. The degrees of association among the variables were assessed through the Chi-square test. After conducting Chi-square analysis, only variables found to be statistically significant were put into the adjusted multiple logistic regression model. Adjusted OR (AOR) and their 95 % CI were used as indicators of the strength of the association.

## Results

### Nutritional status of the elderly forcibly displaced Myanmar nationals

The mean score in MNA@-SF was 10·38 (±3·35) with a minimum of 3 and a maximum of 14. Participants who scored between 0 and 7 points were categorised as ‘malnourished’, 8 and 11 points were categorised as ‘at risk of malnutrition, and more than 11 points indicates normal nutrition status. According to the MNA@-SF, the prevalence of malnutrition among elderly FDMN was 25·3 %, and another 29·1 % were at risk of malnutrition. The remaining 45·8 % were at normal nutritional status.

### Geriatric depression of forcibly displaced Myanmar nationals

The mean score in GDS-15 SF was 5·77 (±2·1) with a minimum of 2 and a maximum of 12. A score of ≥ 5 was used as an indicator for GD. The prevalence of GD was found to be 57·9 %, which is very high.

### Descriptive statistics of demographic factors

The mean age of the study participants was 71·7 (±7·8) years, the minimum age was 60 years and the maximum age was 90 years. The majority of the study participants were aged between 70 and 79 years (41·6 %). The mean BMI was 21·94 kg/m^2^ (±2·6) with a minimum of 15·92 and a maximum of 29·59. The majority of study participants were found to be in the normal BMI range (72·6 %), 23·7 % were underweight and only 3·7 % were found overweight. The proportion of males and females was not significantly different (53·3 % were female and 46·7 % were male). The percentage of educated (able to minimally read and write in their mother tongue) people was very low (28·8 %). Among the participants, 34 % were smokers and another 24·9 % were found to be ex-smokers. The majority of the households (74 %) have at least five members, and more than 70 % have at least five children (Table 1).

**Table 1:** Descriptive statistics of demographic variables and association with geriatric depression, 2021

Variables	All participants, *n* 430		Non-depressed, *n* 181		Depressed, *n* 249		*P* value
	*n*	%	*n*	%	*n*	%	
Age group							
60–69 years	170	39·5	78	45·9	92	52·1	0·02*
70–79 years	179	41·6	80	44·7	99	55·3	
≥80 years	81	18·8	23	28·4	58	71·3	
Sex							
Male	229	53·3	87	38·0	142	62·0	0·07
Female	201	46·7	94	46·7	107	53·2	
Educational status							
Educated	124	28·8	59	47·6	65	52·4	0·14
Uneducated	306	71·2	122	39·9	184	60·1	
Marital status							
Married	315	73·3	125	39·7	190	60·3	0·23
Widowed	95	22·1	47	49·5	48	50·5	
Others (divorced/lost)	20	4·7	9	45·0	11	55·0	
Smoking status							
Smoker	146	34·0	54	37·0	92	63·0	0·24
Ex-smoker	107	24·9	45	42·1	62	57·9	
Non-smoker	177	41·2	82	46·3	95	53·7	
Numbers of member in household							
<5 members	112	26·0	36	32·1	76	67·9	0·01*
≥5 members	318	74·0	145	45·6	173	54·4	
Numbers of children							
<5 children	125	29·1	47	37·6	78	62·4	0·23
≥5 children	305	70·9	134	43·9	171	56·1	

* Statistically significant.

### Association between demographic variables and geriatric depression

The association between demographic variables and GD was analysed by the Chi-square test. Among the demographic variables, there was a statistically significant association between the age of the participants (*P* = 0·02) and the number of family members with GD (Table 1).

### Descriptive statistics of co-morbidities

We have assessed the most common self-reported co-morbidities found among elderly FDMN. Diabetes mellitus was seen in 32·1 % of participants, hypertension in 26·7 % of participants and hypotension in 20 % of participants. In elderly FDMN, various types of skin diseases were found in 28·4 % of the participants. Scabies (11·2 %), taeniasis (7·9 %) and eczema (7·7 %) were the most common skin diseases. COPD was also seen in 16·5 % of participants (Table 2).

**Table 2:** Descriptive status of co-morbidities and association with geriatric depression, 2021

Variables	All participants, *n* 430		Non-depressed, *n* 181		Depressed, *n* 249		*P* value
	*n*	%	*n*	%	*n*	%	
Diabetes mellitus							
No	292	67·9	138	47·3	154	52·7	<0·01*
Yes	138	32·1	43	31·2	95	68·8	
Blood pressure							
Normal	229	53·3	112	48·9	117	51·1	0·01*
Hypotension	86	20·0	27	31·4	59	68·6	
Hypertension	115	26·7	42	36·5	73	63·5	
Skin disease							
No	308	71·6	139	45·1	169	54·9	0·04*
Yes	122	28·4	42	34·4	80	65·5	
Chronic obstructive pulmonary disease							
No	359	83·5	148	41·2	211	58·8	0·41
Yes	71	16·5	33	46·5	38	53·5	
Nutritional status							
Malnourished	109	25·3	34	31·2	75	68·8	<0·01*
Risk of malnutrition	124	28·8	50	40·3	74	59·7	
Normal	197	45·8	97	49·2	100	50·8	

* Statistically significant.

### Association between co-morbidities and geriatric depression

We have also analysed the association between co-morbidities and GD through Chi-square statistics. The presence of co-morbidities such as diabetes mellitus (*P* < 0·01), blood pressure (*P* = 0·01) and skin disease (*P* = 0·04) had a statistically significant association with the increasing prevalence of GD. According to our analysis, GD significantly varies with the nutritional status of the participants. The depression status of malnourished participants was found to be significantly higher compared to normal participants (*P* < 0·01) (Table 2).

### Determination of independent predicators of geriatric depression

Our multiple logistic regression analysis indicates that participants at least 80 years old were at 84 % higher risk of GD (AOR = 1·84, 95 % CI: 1·01, 3·37), and participants who had fewer than five members in their household were at 82 % more risk of GD. Diabetes mellitus (AOR = 1·95, 95 % CI: 1·24, 3·08) and hypotension (AOR = 2·17, 95 % CI: 1·25, 2·78) were also identified as independent predictors of GD. Moreover, malnutrition was also identified as an important predictor of GD (AOR = 1·71, 95 % CI: 1·01, 2·89) (Table 3).

**Table 3:** Multiple logistic regression analysis of statistically significant factors geriatric depression, 2021

Associated factor	AOR	95 % Confidence Interval	*P* value
		Lower CI, Upper CI	
Age (70–79 years)	0·95	0·61, 1·51	0·84
Age (≥80 years)	1·84	1·01, 3·37	0·04*
Members in household (<5 persons)	1·82	1·13, 2·93	0·02*
Diabetes mellitus (yes)	1·95	1·24, 3·08	0·01*
Blood pressure (hypertension)	1·33	0·81, 2·19	0·26
Blood pressure (hypotension)	2·17	1·25, 2·78	0·01*
Skin disease (yes)	1·49	0·94, 2·38	0·09
Nutritional status (malnutrition)	1·71	1·01, 2·89	0·04*
Nutritional status (at risk)	1·29	0·81, 2·08	0·28

* Statistically significant.

## Discussion

Older refugees have a relatively lower ability to meet their basic needs due to their physical and mental disabilities or social impairments^(24)^. We have successfully assessed the nutritional status and depression of elderly FDMN and also determined the self-reported status of other selected co-morbidities. There was a high prevalence of geriatric malnutrition, GD and other selected co-morbidities among FDMN. The increase in the GD proportion was also associated with malnutrition and some other co-morbidities.

The overall prevalence of GD was relatively high (58 %) among FDMN in Bangladesh. That is relatively higher than some previously conducted studies in Bangladesh, which showed the prevalence of GD ranging from 36·9 % to 45 %^(25,26)^. A study conducted among Rohingya refugees in Bangladesh identified a 70 % prevalence of depression and 8·7 % of severe depression^(27)^, and another study identified a 41 % prevalence of depression among the elderly group of those refugees^(28)^.

Historically, malnutrition among elderly refugees has been a problem in several settings^(24)^. According to our analysis, about one-fourth of the elderly FDMN had malnutrition, and another 29·1 % were at risk of malnutrition. A recently conducted study in Myanmar identified a 21·7 % prevalence of malnutrition, and another 59·4 % were at risk for malnutrition^(29)^. Refugees usually face a double burden of malnutrition due to food shortages, unavailability of portable water and inadequate health services^(30)^. Previous data on the Bosnian conflict suggest that elderly people who are more than 60 years old have the highest risk of malnutrition^(31)^. Impairment in physical ability, access to land, meeting basic needs, access to essential services and psychosocial trauma are risk factors for nutritional risk for older refugees^(32)^. Therefore, humanitarian agencies working with refugees should focus on the risk factors of malnutrition, which is itself associated with increasing the burden of co-morbidities^(30)^.

Elderly people are at high risk of common NCD and various skin diseases. On the other hand, refugees, socially disadvantaged people and vulnerable groups of people are at higher risk of NCD. In our study, the prevalence of prediabetes and diabetes mellitus was 32·1 % in elderly FDMN. Hypertension was seen in 26·7 % of participants and hypotension in 20 % of participants. A study conducted in India observed the prevalence of Hypertension and Diabetes mellitus among the elderly to be 54·5 % and 14·6 %, respectively^(33)^. Another recent study has identified a moderately high prevalence of NCD risk factors among refugees in Cox’s Bazar^(34)^. The high prevalence of NCD and their risk factors indicate gaps in the policies to control NCD among refugees, which need to be included in the policy discussions.

A sound idea regarding the skin diseases of the refugee population is important for planning and decision-making for the healthcare of this vulnerable group. A study conducted among FDMN observed a high prevalence of various skin diseases, especially fungal skin infections^(35)^. Our analysis also observed that 28·4 % of the elderly FDMN had a skin infection. Scabies (11·2 %), taeniasis (7·9 %) and eczema (7·7 %) were the most common skin diseases. COPD was also seen in 16·5 % of participants. Previous studies have also observed a high prevalence of COPD among the FDMN community of Bangladesh^(36)^.

Our analysis has also identified malnutrition as a statistically significant predictor of GD and associated with a 70 % increased risk of GD. Due to the psychological status of elderly people, it is common to catch depression, and malnutrition makes the situation more vulnerable^(37)^. A recent study in Bangladesh also identified a significantly higher risk of GD among malnourished elderly people^(38)^. Therefore, it is high time to do a vast nutritional assessment focusing on the FDMN community and develop an interventional model to improve the situation. These will pave the pathway to controlling NCD among FDMN and improving the mental health and overall quality of life of refugees.

Previous studies suggest that an increase in age is significantly associated with depression, supporting our study findings^(28)^. Our analysis indicates participants who were at least 80 years old were at 84 % higher risk of GD compared to the 60–69 years aged group, and participants who had fewer than five members in their household were at 82 % higher risk of GD. Mental health is one of the most important predictors of quality of life, which is especially important for elderly people^(39)^. Therefore, NCD policies should include a separate policy for mental health, with a priority on geriatric depression.

Diabetes mellitus, hypotension and skin diseases are some of the significant predictors of GD. A high prevalence of these NCD and their risk factors in the Rohingya refugee camps makes it essential to support the formulation of NCD policies for Rohingya refugees in Bangladesh. The association between diabetes mellitus and depression has already been established, and the impact is also similar among elderly patients^(40)^. However, the association between hypotension and GD is controversial, though we have identified it as an independent predictor^(41)^. The association between depression and skin infection is also established, which also contributes to the psychosocial impairment of elderly people^(42)^. These established associations will also help policymakers develop NCD policies for refugees in Bangladesh and an intervention model to control NCD in Rohingya camps.

### Limitations

Besides the important findings mentioned above, our study was completed with a few limitations. Firstly, due to the unavailability of funding, we were absolutely dependent on previous test reports, medical documents or discussions with family members for the identification of co-morbidities among the study participants. Secondly, due to the nature of the cross-sectional study design, it is difficult to derive or interpret any causal relationships. Thus, this study gave us information about the prevalence of GD among FDMN in the Cox’s Bazar refugee camps of Bangladesh and its association with the status of geriatric malnutrition and the presence of co-morbidities. Moreover, we have developed a questionnaire in the local Bengali language, which is very close to the FDMN’s language. As there were no experts in Rohingya in our team, we were dependent on the interpreter if any respondents were unable to understand any part of the questionnaire and collect their responses. During data collection, a higher proportion of females declined to participate compared to males, which might lead to selection bias.

### Conclusions

The present study has identified a very high prevalence of GD among elderly FDMN in Bangladesh. The nutritional assessment also revealed a high prevalence of malnutrition, an independent predictor of GD. Elderly FDMN are already in a vulnerable situation due to their limitations, and the presence of depression with nutritional deficiencies made the situation worse. A number of other important factors are also associated with a high prevalence of depression and other worse situations. The planners and policymakers should consider those factors to provide elderly FDMN with a livable situation. Based on our observations, it is recommended to ensure equity for elderly FDMN regarding health services, basic needs and mental health support. To improve the quality of life, it is also essential to ensure screening for GD and nutritional status among elderly FDMN to prevent worse health situations. Corresponding authorities would arrange special support centres for elderly FDMN in Bangladesh. To improve the quality of life, it is also essential to ensure screening for GD and nutritional status.
